# Supplementary material for: Mental health recovery for survivors of modern slavery: grounded theory study protocol
Source: BMJ Open. 2020 Nov 19;10(11):e038583. doi: 10.1136/bmjopen-2020-038583 (PMC7678374; doi:10.1136/bmjopen-2020-038583)
Supplement: Supplementary data [file bmjopen-2020-038583supp001.pdf]

•Distress

- A participant indicates that they are experiencing distress. **OR**
- A participant exhibits behaviours suggestive that the interview is causing distress. For example crying, shaking, agitation.

•Stage 1 response

- The researcher stops the interview.
- The participant is offered immediate support, time out etc..
- The researcher asks the participant how they are feeling, what their thoughts are and whether they feel safe.

•Review

- If the participant feels able to carry on resume the interview.
- If the participant is unable to carry on go to stage 2 response.

•Stage 2 response

- Discontinue interview.
- The participant is encouraged to seek support from their usual contact points. This could be a caseworker/therapist at an NGO, their GP, a local authority worker or mental health team. **OR**
- With consent offer for a member of the research team to make contact with the participant usual support system. **OR**
- With consent seek advice or further support from the participants main contact point.

•Follow up

- If participant consents follow up with a courtesy phone call. **OR**
- Encourage the participant to call if s/he experiences increasing levels of distress in the hours/days post interview.
- All participants to be given an information sheet with relevant organisations contact details and places for support (translated if necessary).

•Distress Protocol 1: The protocol for managing participant distress in the context of a research interview

•Pre-data collection

- •The potential physical and psychological impact on the researcher of the participants description of their life events should be considered.
- •In collaboration with the PI and wider research team a decision should be made as to how many interviews could be undertaken in a week.
- •The potential for emotional exhaustion should be acknowledged.
- •A mechanism for debriefing and clinical supervision should be established to provide support during data collection and analysis.

• Data collection stage

- •Consider where interviews should take place to ensure support is available . Also take into account the University of Nottingham Lone Worker Policy to ensure safety.
- •Debriefing to be available with the PI or another member of the research team immediately after an interview has been completed if required.
- •Monthly clinical supervision to be arranged with a member of staff external to the research team to facilitate reflection and processing of events.
- •Encourage the researcher to keep a reflective diary which records thoughts and feeling which could become part of research fieldnotes if appropriate.

•Analysis

- •For the researcher to be aware that engaging in data analysis of potentially distressing material could be emotionally triggering.
- •Schedule monthly clinical supervision during this period of time to discuss emerging situations, encourage reflection and processing of events.
- •Access debriefing as required with a member of the research team.
- •Encourage the researcher to keep a reflective diary which records thoughts and feeling which could become part of research fieldnotes if appropriate.

•Follow up

- •Encourage the researcher to seek counselling and other support in addition to debriefing and clinical supervision should s/he experience increased distress as a result of engagement in research activities.

•Distress Protocol 2: The protocol for managing distress in the context of research interview management.

•Pre-data collection

- The transcriber should be considered in any research proposal, with a clear indication of how this person will be provided with an emotionally safe working environment which also maintains the quality of the research product.

•Ethical •considerations

- Consideration is given to the support needs of the transcriber as part of the ethical review process.
- The transcriber should be informed of the nature of the research and the type of data to be collected.

•Pre- •transcription

- The transcriber is alerted prior to commencing transcription of any potentially challenging or difficult interviews.
- The transcriber has access to regular debriefing with the PI or another member of the research team should this be required.

•During transcription

- Prompt access to an appropriate person for crisis debriefing to be facilitated should this be required.
- The transcriber is able to terminate their involvement in the transcription process (either of an individual interview or from the whole project) if deemed necessary with appropriate support given to resolve any personal issues that arise as consequence of this work.
- Encourage the transcriber to keep a reflective diary to record their thoughts and feelings which may become part of fieldnotes if appropriate.

•Follow up

- If the transcriber consents, follow up with a courtesy call. **OR**
- Encourage the transcriber to call if s/he experiences increased distress in the hours/days following transcription.

•Distress Protocol 3: The protocol for managing distress in the context of research interview transcription.

- Pre-data collection
  - The interpreter should be considered in any research proposal, with a clear indication of how this person will be provided with an emotionally safe working environment which also maintains the quality of the research product.

- Ethical
- considerations
  - Consideration is given to the support needs of the interpreter as part of the ethical review process.
  - The interpreter should be informed of the nature of the research and the type of data to be collected.

- Data collection
  - The interpreter is alerted to any potentially challenging interviews which have been scheduled in advance.
  - Debriefing to be available with the PI or another member of the research team immediately after an interview has been completed if required.
  - Encourage the interpreter to keep a reflective diary which records thoughts and feeling which could become part of research fieldnotes if appropriate.

- Follow up
  - If the interpreter consents, follow up with a courtesy call. **OR**
  - Encourage the interpreter to call if s/he experiences increased distress in the hours/days following transcription.

- Distress Protocol 4: The protocol for managing distress in the context of research interview interpretation.

•Supporting References

- Draucker CB, Martsolf DS & Poole C
  •(2009) Developing distress protocols for
  •research on sensitive topies. **Archives**
  •**of Psychiatric Nursing** 23 (5) 343-350.
- Gregory D, Russell C & Phillips L (1997)
  •Beyond textual perfection: transcribers
  •as vulnerable persons. **Qualitative**
  •**Health Research** 7 (2) 294-300.
- Haigh C & Witham G (2015) **Distress**
  •**Protocol for Qualitative Data**
  •**Collection.** Manchester Metropolitan
  •University.
- McCosker H, Barnard A & Gerber R
  •(2001) Undertaking sensitive research:
  •Issues and strategies for meeting the
  •safety needs of all. **Forum: Qualitative**
  •**Social Research** 2 (1).
