# Supplementary material for: Mental health recovery for survivors of modern slavery: grounded theory study protocol
Source: BMJ Open. 2020 Nov 19;10(11):e038583. doi: 10.1136/bmjopen-2020-038583 (PMC7678374; doi:10.1136/bmjopen-2020-038583)
Supplement: Author's manuscript [file bmjopen-2020-038583.draft_revisions.pdf]

BMJ Open is committed to open peer review. As part of this commitment we make the peer review history of every article we publish publicly available.

When an article is published we post the peer reviewers' comments and the authors' responses online. We also post the versions of the paper that were used during peer review. These are the versions that the peer review comments apply to.

The versions of the paper that follow are the versions that were submitted during the peer review process. They are not the versions of record or the final published versions. They should not be cited or distributed as the published version of this manuscript.

BMJ Open is an open access journal and the full, final, typeset and author-corrected version of record of the manuscript is available on our site with no access controls, subscription charges or pay-per-view fees (http://bmjopen.bmj.com).

If you have any questions on BMJ Open's open peer review process please email info.bmjopen@bmj.com

Mental Health Recovery for Survivors of Modern Slavery: Grounded Theory Study Protocol

Journal:	*BMJ Open*
Manuscript ID	bmjopen-2020-038583
Article Type:	Protocol
Date Submitted by the Author:	18-Mar-2020
Complete List of Authors:	Wright, Nicola; University of Nottingham, School Health Sciences<br>Hadziosmanovic, Emina; University of Nottingham, School of Health Sciences<br>Dang, Minh; University of Nottingham, School of Politics and International Relations; Survivor Alliance<br>Bales , Kevin; University of Nottingham, School of Politics and International Relations<br>Brookes, Caroline; Nottinghamshire Healthcare NHS Foundation Trust, Emergency Preparedness Resilience & Response (EPRR)<br>Jordon, Melanie; University of Nottingham, School of Sociology and Social Policy<br>Slade, Mike; University of Nottingham School of Health Sciences, Research Advisory Board, Lived Experience ; Survivor Alliance
Keywords:	Adult psychiatry < PSYCHIATRY, QUALITATIVE RESEARCH, Anxiety disorders < PSYCHIATRY

SCHOLARONE™
Manuscripts

I, the Submitting Author has the right to grant and does grant on behalf of all authors of the Work (as defined in the below author licence), an exclusive licence and/or a non-exclusive licence for contributions from authors who are: i) UK Crown employees; ii) where BMJ has agreed a CC-BY licence shall apply, and/or iii) in accordance with the terms applicable for US Federal Government officers or employees acting as part of their official duties; on a worldwide, perpetual, irrevocable, royalty-free basis to BMJ Publishing Group Ltd ("BMJ") its licensees and where the relevant Journal is co-owned by BMJ to the co-owners of the Journal, to publish the Work in this journal and any other BMJ products and to exploit all rights, as set out in our licence.

The Submitting Author accepts and understands that any supply made under these terms is made by BMJ to the Submitting Author unless you are acting as an employee on behalf of your employer or a postgraduate student of an affiliated institution which is paying any applicable article publishing charge ("APC") for Open Access articles. Where the Submitting Author wishes to make the Work available on an Open Access basis (and intends to pay the relevant APC), the terms of reuse of such Open Access shall be governed by a Creative Commons licence – details of these licences and which Creative Commons licence will apply to this Work are set out in our licence referred to above.

Other than as permitted in any relevant BMJ Author's Self Archiving Policies, I confirm this Work has not been accepted for publication elsewhere, is not being considered for publication elsewhere and does not duplicate material already published. I confirm all authors consent to publication of this Work and authorise the granting of this licence.

For peer review only

Title: Mental Health Recovery for Survivors of Modern Slavery: Grounded Theory Study Protocol

Authors: Wright N, Hadziosmanovic E, Dang M, Bales K, Brookes C, Jordan M, Slade M and the Lived Experience Research Advisory Board.

Affiliations:

Dr Nicola Wright, University of Nottingham

Dr Emina Hadziosmanovic, University of Nottingham

Miss Minh Dang, University of Nottingham and Survivor Alliance

Professor Kevin Bales, University of Nottingham

Mrs Caroline Brookes, Nottinghamshire Healthcare NHS Foundation Trust

Dr Melanie Jordan, University of Nottingham

Professor Mike Slade, University of Nottingham

Lived Experience Research Advisory Board, Survivor Alliance

Corresponding Author:

Dr Nicola Wright, School of Health Sciences, University of Nottingham

Address: Queen’s Medical Centre, Nottingham, NG7 2UH

Email: Nicola.wright@nottingham.ac.uk

For peer review only

Abstract

Introduction

Slavery and human trafficking are global crimes involving the violation of human rights. Epidemiological studies show high levels of mental health need and poor provision of appropriate support for survivors. What mental health recovery means and how it could be promoted in this population is under-researched.

Methods and Analysis

A grounded theory study based on individual interviews will be undertaken. Survivors from across the UK will be identified and recruited from Non-Governmental Organisations (NGOs) and via social media. As per grounded theory methodology, data collection and analysis will be undertaken concurrently and recruitment will continue until theoretical saturation is reached. It is anticipated that approximately 30 participants will be required. Interviews will be audio recorded, transcribed verbatim and uploaded to NVivo 11. The constant comparative method will be used to analyse the data, in order to produce a theoretical framework for mental health recovery which is grounded in the experiences of survivors.

Ethics and Dissemination

Ethical approval has been obtained from the Faculty of Medicine and Health Sciences Ethics Committee at the University of Nottingham. The findings of the study will be disseminated to academic, professional, and survivor-based audiences to inform future policy developments and the provision of mental health recovery support to this population .

Article Summary

Strengths and limitations of this study

- The study will allow an in-depth understanding of what mental health recovery means to survivors of modern slavery.
- The study will generate knowledge in the form of a theoretical framework that will facilitate the development of practice recommendations and interventions to promote mental health.
- The study has been co-developed with survivors of modern slavery and a lived experience advisory board will facilitate the involvement of survivor voices throughout the research process.
- The theoretical framework will be developed based on a UK sample, and international variations in how concepts such as mental health and recovery are understood may limit its cross-cultural validity.

For peer review only

Introduction

Slavery and human trafficking are crimes involving the violation of human rights and refer to exploitative situations where an individual cannot refuse or leave due to threats, coercion or abuse of power[1,2]. Activities involving slavery include forced labour exploitation, forced sexual exploitation, forced marriage and servitude. It is estimated that approximately 40.3 million people are currently in slavery worldwide[3]. Predicting the future prevalence of slavery is problematic. Globally, the number of vulnerable migrants at risk of trafficking is increasing, with an estimated 65 million people forcibly displaced globally in 2015[4].

In the United Kingdom (UK), research conducted by the Home Office in 2014[5] estimated that there were between 10,000 and 13,000 victims of modern slavery in the UK. However, the Home Office in 2017 has argued that this is the "*tip of the iceberg*"[6]. Due to fear of arrest, deportation or ill treatment by the police, many survivors choose not to access the support provided through official channels, e.g. the National Referral Mechanism in the UK[7]. Therefore, official figures do not take into account the unknown numbers of people who are not reported or who continue to live in slavery. Despite these limitations, official statistics do highlight that more people are being freed and are therefore in need of support. They also demonstrate that this is a diverse population, with UK survivors originating from 130 different countries including the UK, Albania, Vietnam, China, Romania, Sudan, Eritrea, Nigeria, India, and Pakistan[8].

Modern slavery (in the UK context this term encompasses both slavery and human trafficking) presents a significant public health concern, and disproportionately affects vulnerable individuals, such as young people, migrants and those living in poverty[9]. Survivors have often experienced extreme violence and psychological abuse[10]. Addressing the mental health needs of this population is therefore now part of anti-trafficking policies in the UK such as the Modern Slavery Act[11], as well as internationally, for example in the Palmero Protocols[12]. Yet, the provision of evidence-based mental health support for this population is one of the largest gaps in both the national and global anti-slavery response[13].

The literature that is available, has predominantly focussed on the mental health needs of women, including those trafficked for sexual exploitation in South or South East Asia and to a lesser extent Europe[14]. Abas and colleagues [15] identified that for women who returned home from the UK to Moldova after being sex trafficked, 55% continued to meet the diagnostic criteria for depression and PTSD six months after their return. Data from high income countries (including the UK), for men and those who have experienced forced labour, is sparse[14]

The limited evidence available from the UK highlights a complex picture in relation to the mental health needs of survivors. High prevalence rates of depression, anxiety, PTSD and suicidal ideation have been identified[7], as well as unmet needs in relation to hearing voices. [7]. For young people who have been trafficked, ADHD and adjustment disorders are common[16]. Whilst these studies have highlighted the level of mental health need within the population, what is not clear is how

For peer review only - http://bmjopen.bmj.com/site/about/guidelines.xhtml

individuals perceive their mental health; what resources they have to overcome their adverse experiences; and what support or interventions would be helpful to facilitate growth and recovery(13).

Post Traumatic Growth (PTG) is defined as the individuals' psychological development after an adverse incident(17). Whilst traditional theories of resilience emphasise the importance of a return to normal after a traumatic event, PTG recognises that adversity can lead to significant changes, some of which may be experienced as positive changes(17). Rather than a problem or illness orientated position, PTG emphasises the active role of the individual in processing the event and constructing a new identity in the aftermath of trauma(18). More recent work has started to apply the theory of PTG to clinical populations, for example those with psychosis(19). Through this personal growth process, the strengths and resources of the individual are emphasised, and positive adaptions learnt.

Within this context, modern slavery survivors are seen as active participants in their care, with resources and strengths which can be utilised for mental health recovery(20). This can include factors within the individual, such as coping strategies, and also those external to them, for example social networks. Recovery in this context is therefore a subjective experience and has been defined as "a deeply personal, unique process of changing one's attitudes, values, feelings, goals, skills and/or roles" and as "a way of living a satisfying, hopeful and contributing life even within the limitations caused by illness"(21). It ultimately means that individuals are supported to live as well as possible.

Recovery has become the underpinning discourse for mental health service provision globally(22) and in England(23). A key insight from this perspective is that living well involves less emphasis on symptom amelioration, and a stronger focus on addressing psychological and social needs, supporting self-management and building individual and community resilience(24). This understanding is relevant to work with survivors of modern slavery, who may have mental health symptoms (e.g. those related to trauma) and also a range of other psychological (e.g. self-identity), social (e.g. anticipated and experienced discrimination) and cultural challenges (e.g. dislocation).

The aim of this study is to develop a theoretically informed understanding of mental health recovery for survivors of modern slavery. The objectives are: (1) To understand the mental health needs and strengths of modern slavery survivors; (2) To explore with modern slavery survivors the concept of mental health recovery; what it means to them; and how it could be promoted; and (3) To construct a theoretically informed framework for mental health recovery, based on the experiences of modern slavery survivors, which can inform a future intervention.

Methods and Analysis

Design

A qualitative, grounded theory study design based on individual, semi-structured interviews with modern slavery survivors is proposed. Widely used in health and social sciences, grounded theory is an inductive methodology which facilitates the development of a theoretical framework grounded in the experiences of participants(25).

For peer review only - http://bmjopen.bmj.com/site/about/guidelines.xhtml

Setting

Participants will be recruited from relevant Non-Governmental Organisations (NGOs) and through social media from across the UK.

Sample

Eligibility criteria:

(1) Self-identify or be identified by an NGO as a survivor of modern slavery. Self-identifying involves describing being in situations or having experiences that align with modern slavery, even if the term 'modern slavery' is not used by the participant.
(2) Be currently in the UK
(3) Be aged over 18 years.
(4) Have exited slavery for more than 12 months.
(5) Be able to participate in an interview process conducted in English, either with or without the assistance of an interpreter.
(6) Be able and willing to discuss issues related to mental health/distress.
(7) A formal diagnosis of a mental health problem is not required, although potential participants should have one of: (a) self-reported clinical diagnosis; (b) recent primary or secondary mental health service use; OR (c) self-reported recent experience of mental distress.

Procedures

Aligned with the study design, theoretical sampling will be used. This is an iterative process whereby data collection and analysis occur simultaneously, allowing insights from preliminary analysis to inform the recruitment of future participants. This will occur until data saturation is reached, i.e. no new insights or themes are identified[25]. After an initial sample of participants have been recruited and their data analysed, future sampling will address potentially any of the following:

(1) Gaps in relation to participant demographics (for example gender, age, ethnicity, experience of a particular form of slavery);
(2) Emergent theoretical concepts, for example to understand or elucidate a particular insight in more detail;
(3) Any explanatory gaps between the concepts identified, for example to demonstrate the relationship between two different concepts.

If necessary, theoretical sampling will be supplemented with snowball sampling techniques, whereby participants are asked to identify from within their networks other individuals to approach for interview. The nature of theoretical sampling and achieving data saturation means that determining in advance the precise sample size is problematic. Based on previous grounded theory studies, 30 interviews is likely to be sufficient to achieve saturation[26, 27]. Therefore, this study will recruit 30 modern slavery survivors or until data saturation is achieved

In-depth, semi-structured interviews will be conducted with participants. Where necessary, independent interpreters will be used during the interviews and written materials translated. The interview schedules were developed in collaboration with the Lived Experience Advisory Board and informed by the World Health Organisation recommendations for interviewing trafficked women[28]. As per the iterative nature of

For peer review only - http://bmjopen.bmj.com/site/about/guidelines.xhtml

grounded theory, the interview schedules will continue to evolve during the course of the project to address emergent concepts and ideas with participants.

Recruitment of participants will take place via relevant NGOs who provide support to survivors of modern slavery, and by advertising the study widely through social media. The research team will work with the organisations to distribute study information and identify people who meet the eligibility criteria. Individuals who are interested in participating in the study will be asked to contact the research team by either phone or email. After explaining the study purpose, including the voluntary and anonymous nature of participation, and distributing the information sheet, informed written consent will be obtained. The interviews (approximate duration 60 minutes) will be audio-recorded and transcribed verbatim. They will be conducted at a time of the participants choosing, whenever possible as a face-to-face interview in a place which assures the participant of safety, privacy and comfort (and by phone where not possible).

Data Analysis

Interview transcripts will be uploaded to NVivo 11 software for analysis. The constant comparative method will be used to generate successively more abstract concepts and theories through the inductive coding of individual transcripts[(29)]. The codes identified will then be compared against those generated in other transcripts. This will identify both consistency and any divergence in the theoretical framework being constructed.

Patient and Public Involvement

Patient and public involvement is a core component of the project. As per guidelines[(31)] survivors are engaged in the five key stages of the research process:

(1) Design of the research.
(2) Development of the grant application/pre-protocol work.
(3) Undertaking or managing the research.
(4) Analysis of data.
(5) Dissemination of findings.

A survivor led, lived experience research advisory board has been developed in collaboration with the Survivor Alliance (www.survivoralliance.org), a non-profit organisation that unites and empowers survivors of slavery and human trafficking worldwide. The role of the advisory board will be to provide a survivor perspective on all aspects of the study, including language and format of consent forms and information sheets, recruitment strategy, data analysis and dissemination. The group will meet six times during the course of the project.

Ethics and Dissemination

Ethical approval for the study was obtained from the Faculty of Medicine and Health Sciences, University of Nottingham in December 2019 (reference number 436-1912). The main ethical concern of the project is the potential to cause emotional or psychological distress to participants, if they recount their traumatic experiences. The project aims to minimise this by not specifically asking questions relating to the original

For peer review only - http://bmjopen.bmj.com/site/about/guidelines.xhtml

trauma or trafficking experience. Instead, the interview will focus on mental health recovery and what this means to individuals. It is acknowledged that individuals may choose to recount their difficult experiences and if they do so, the distress protocol (Supplementary Information 1) will be adhered to.

Participants will remain anonymous, their names (and any other identifiable details) will be replaced by a pseudonym at the point of transcription. Individuals' identities will not be disclosed in the final report or any subsequent publications. Secure electronic and paper based filing systems for both the recordings and transcriptions will be established. All study related data will be kept for a minimum of 7 years after the study end date.

The findings of the research will be disseminated to academic, professional, survivor, and policy audiences via written publication, verbal presentations and knowledge sharing events.

Strengths and Limitations

Modern slavery survivors are not a homogenous population and the recruitment strategy may limit the relevance of the findings to particular sub-groups. Cultural variation in how concepts such as mental health and recovery are interpreted may also impact on the cultural validity of the resulting framework. For example, in some cultures admitting to mental health difficulties may be stigmatising and so individuals may be unwilling to discuss their experiences. However, the voices of survivors are currently lacking within the mental health sphere and this study offers the opportunity to start to develop a framework for understanding modern slavery which is based on experiential knowledge rather than expert opinion.

Conclusion

The need to provide appropriate and timely mental health support to survivors is highlighted in a number of national and international policies. Research indicates that survivors have a complex array of mental health problems which are not currently being met by the services which are available. However, there is a lack of qualitative research into the complex nature of survivors' mental health experiences, what strengths and resources individuals have and their understanding of mental health recovery. This study will explore with survivors what recovery means to them and how they can be supported to live as well as possible. Generating such knowledge will facilitate the development of a theoretical framework of mental health recovery for survivors of modern slavery. This will provide an empirically-based theoretical foundation for develop and modifying intervention to support the recovery of individuals with lived experience of modern slavery.

For peer review only - http://bmjopen.bmj.com/site/about/guidelines.xhtml

References

1) Williamson, V, et al. Responding to the needs of trafficked people: A qualitative study of professionals in England and Scotland. **Health and Social Care in the Community** 2020; 28: 173 – 181.

2) International Labour Organisation, **Methodology of the global estimates of modern slavery: Forced labour and forced marriage.** The International Labour Office: Geneva. 2017.

3) Global Slavery Index, **The Global Slavery Index 2018.** [online] Accessed on: 3rd March 2020 at www.globalslaveryindex.org.

4) United Nations Office on Drugs and Crime, **Global Report on Trafficking in Persons.** United Nations: New York. 2016.

5) Silverman, B, **Modern Slavery: An Application of Multiple Systems Estimation.** Home Office: London. 2014.

6) Home Office, **2017 UK Annual Report on Modern Slavery.** [online] Accessed on: 3rd March 2020 at https://www.gov.uk/government/publications/2017-uk-annual-report-on-modern-slavery.

7) Oram, S, *et al.* Human trafficking and health: A survey of male and female survivors in England. **American Journal of Public Health** 2016; 106: 6 1073-1078.

8) Home Office, **2019 UK Annual Report on Modern Slavery.** HMSO: London. 2019.

9) Zimmerman, C. and Kiss, L. Human trafficking and exploitation: A global health concern. **PLOS Medicine** 2017; 14: 11 e1002437.

10) International Organisation of Migration, **Report of the Counter-Trafficking Unit: Return and Reintegration Project.** International Organisation for Migration: Brussels. 2002.

11) The UK Government, **Modern Slavery Act.** HMSO: London. 2015.

12) United Nations, **Protocol to Prevent, Suppress and Punish Trafficking in Persons, Especially Women and Children, Supplementing the United Nations Convention Against Transnational Organised Crime**. New York: UNODC. 2000.

13) Katona, C, *et al.* **Addressing Mental Health Needs in Survivors of Modern Slavery: A Critical Review and Research Agenda.** Helen Bamber Foundation: London. 2015.

14) Oram, S, *et al.* International law, national policymaking and the health of trafficked people in the UK. **Health and Human Rights** 2011; 13 (2): E3-16.

For peer review only - http://bmjopen.bmj.com/site/about/guidelines.xhtml

15) Abas, M, *et al.* Risk factors for mental disorders in women survivors of human trafficking: A historical cohort study. **BMC Psychiatry** 2013; 13: 204.

16) Stanley, N, *et al*. The health needs and healthcare experiences of young people trafficked into the UK. **Child Abuse and Neglect** 2016; 59: 100-110.

17) Tedeschi, RG. and Calhoun, LG. **Posttraumatic Growth: Conceptual Foundation and Empirical Evidence.** Lawrence Erlbaum Associates: Philadelphia. 2004.

18) Carver, C. Resilience and thriving: Issue, models and linkages. **Journal of Social Issues.** 2010; 54: 245-266.

19) Slade, M, *et al.* Post-traumatic growth in mental health recovery: qualitative study of narratives. **British Medical Journal.** 2019; Doi:10.1136/bmjopen-2019-029342.

20) Repper, J. and Perkins, R. **Social Inclusion and Recovery: A Guide for Mental Health Practice.** Bailiere Tindall: London. 2003.

21) Anthony, WA. Recovery from mental illness: The guiding vision of the mental health system in the 1990s. **Psychosocial Rehabilitation Journal** 1993; 16: 11-23.

22) World Health Organisation **Mental Health Action Plan 2013-2020.** WHO Press: Geneva. 2013.

23) Department of Health **No Health without Mental Health: A Cross Government Mental Health Outcomes Strategy for All Ages.** Department of Health: London. 2011.

24) Slade, M, *et al.* Uses and abuses of recovery: Implementing recovery-orientated practices in mental health systems. **World Psychiatry** 2014; 13: 12-20.

25) Charmaz, K. **Constructing Grounded Theory.** 2nd Edition. SAGE: New York. 2014.

26) Dworkin, SL. Sample size policy for qualitative studies using in-depth interviews. **Archives of Sexual Behaviour** 2012; 41: 6 1319-1320

27) Palmquist, L, *et al.* Protocol: A grounded theory study of ‘recovery’: Perspectives of adolescent users of mental health services. **BMJOpen** 2017; 7:e015161. doi: 10.1136/bmjopen-2016-01516

28) Zimmerman, C. **WHO Ethical and Safety Recommendations for Interviewing Trafficked Women.** WHO Press: Geneva. 2003.

29) Glaser, BG. and Strauss, A. **The Discovery of Grounded Theory**. Aldine: Chicago. 1967.

For peer review only - http://bmjopen.bmj.com/site/about/guidelines.xhtml

30) Leamy, ME, *et al.* A conceptual framework for personal recovery in mental health: systematic review and narrative synthesis. **British Journal of Psychiatry** 2011; 199: 445-452.

31) NIHR **Patient and Public Involvement in Health and Social Care Research: A Handbook for Researchers.** NIHR: London. 2014.

Contribution

NW and MD conceived of the research idea and with KB, CB, MJ and MS designed the study and secured funding. EH wrote the initial draft of this paper. All authors have read, edited and revised the paper.

Funding Statement

This study is funded by the National Institute for Health Research (NIHR) [Research For Patient Benefit, project reference PB-PG-1217-20036]. Mike Slade acknowledges the support of Center for Mental Health and Substance Abuse, University of South-Eastern Norway and the NIHR Nottingham Biomedical Research Centre. The views expressed are those of the authors and not necessarily those of the NIHR or the Department of Health and Social Care.

Competing Interests Statement

There are no declarations of competing interests

Patient Consent for Publication

Not required.

Ethics Approval

The study has been approved by the Faculty of Medicine and Health Sciences Ethics Committee at the University of Nottingham.

For peer review only

For peer review only - http://bmjopen.bmj.com/site/about/guidelines.xhtml

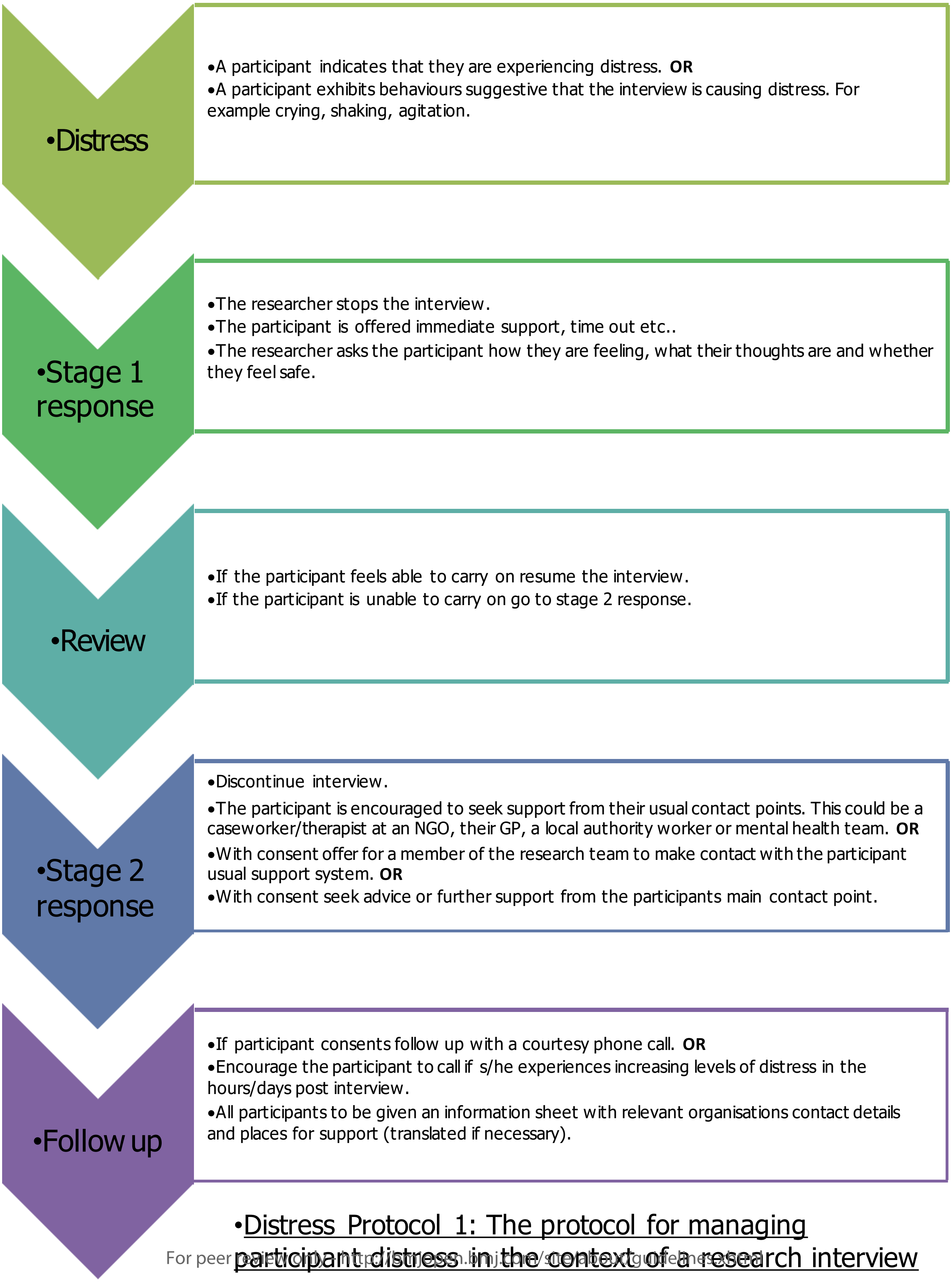

•Distress Protocol 1: The protocol for managing participant distress in the context of a research interview

•Pre-data collection

- •The potential physical and psychological impact on the researcher of the participants description of their life events should be considered.
- •In collaboration with the PI and wider research team a decision should be made as to how many interviews could be undertaken in a week.
- •The potential for emotional exhaustion should be acknowledged.
- •A mechanism for debriefing and clinical supervision should be established to provide support during data collection and analysis.

• Data collection stage

- •Consider where interviews should take place to ensure support is available . Also take into account the University of Nottingham Lone Worker Policy to ensure safety.
- •Debriefing to be available with the PI or another member of the research team immediately after an interview has been completed if required.
- •Monthly clinical supervision to be arranged with a member of staff external to the research team to facilitate reflection and processing of events.
- •Encourage the researcher to keep a reflective diary which records thoughts and feeling which could become part of research fieldnotes if appropriate.

•Analysis

- •For the researcher to be aware that engaging in data analysis of potentially distressing material could be emotionally triggering.
- •Schedule monthly clinical supervision during this period of time to discuss emerging situations, encourage reflection and processing of events.
- •Access debriefing as required with a member of the research team.
- •Encourage the researcher to keep a reflective diary which records thoughts and feeling which could become part of research fieldnotes if appropriate.

•Follow up

- •Encourage the researcher to seek counselling and other support in addition to debriefing and clinical supervision should s/he experience increased distress as a result of engagement in research activities.

•Distress Protocol 2: The protocol for managing distress in the context of research interview management.

For peer review only - http://bmjopen.bmj.com/site/about/guidelines.xhtml

•Pre-data collection

•The transcriber should be considered in any research proposal, with a clear indication of how this person will be provided with an emotionally safe working environment which also maintains the quality of the research product.

•Ethical •considerations

•Consideration is given to the support needs of the transcriber as part of the ethical review process.

•The transcriber should be informed of the nature of the research and the type of data to be collected.

•Pre- •transcription

•The transcriber is alerted prior to commencing transcription of any potentially challenging or difficult interviews.

•The transcriber has access to regular debriefing with the PI or another member of the research team should this be required.

•During transcription

•Prompt access to an appropriate person for crisis debriefing to be facilitated should this be required.

•The transcriber is able to terminate their involvement in the transcription process (either of an individual interview or from the whole project) if deemed necessary with appropriate support given to resolve any personal issues that arise as consequence of this work.

•Encourage the transcriber to keep a reflective diary to record their thoughts and feelings which may become part of fieldnotes if appropriate.

•Follow up

•If the transcriber consents, follow up with a courtesy call. **OR**

•Encourage the transcriber to call if s/he experiences increased distress in the hours/days following transcription.

•Distress Protocol 3: The protocol for managing distress in the context of research interview transcription.

For peer review only - http://bmjopen.bmj.com/site/about/guidelines.xhtml

•Pre-data collection

•The interpreter should be considered in any research proposal, with a clear indication of how this person will be provided with an emotionally safe working environment which also maintains the quality of the research product.

•Ethical •consideration s

•Consideration is given to the support needs of the interpreter as part of the ethical review process.

•The interpreter should be informed of the nature of the research and the type of data to be collected.

•Data collection

•The interpreter is alerted to any potentially challenging interviews which have been scheduled in advance.

•Debriefing to be available with the PI or another member of the research team immediately after an interview has been completed if required.

•Encourage the interpreter to keep a reflective diary which records thoughts and feeling which could become part of research fieldnotes if appropriate.

•Follow up

•If the interpreter consents, follow up with a courtesy call. **OR**

•Encourage the interpreter to call if s/he experiences increased distress in the hours/days following transcription.

•<u>Distress Protocol 4: The protocol for managing distress in the context of research interview interpretation.</u>

For peer review only - http://bmjopen.bmj.com/site/about/guidelines.xhtml

•Supporting References

- Draucker CB, Martsolf DS & Poole C
  - (2009) Developing distress protocols for
  - research on sensitive topies. **Archives**
  - **of Psychiatric Nursing** 23 (5) 343-350.
- Gregory D, Russell C & Phillips L (1997)
  - Beyond textual perfection: transcribers
  - as vulnerable persons. **Qualitative**
  - **Health Research** 7 (2) 294-300.
- Haigh C & Witham G (2015) **Distress**
  - **Protocol for Qualitative Data**
  - **Collection.** Manchester Metropolitan
  - University.
- McCosker H, Barnard A & Gerber R
  - (2001) Undertaking sensitive research:
  - Issues and strategies for meeting the
  - safety needs of all. **Forum: Qualitative**
  - **Social Research** 2 (1).

BMJ Open

Mental Health Recovery for Survivors of Modern Slavery: Grounded Theory Study Protocol

Journal:	*BMJ Open*
Manuscript ID	bmjopen-2020-038583.R1
Article Type:	Protocol
Date Submitted by the Author:	28-Jul-2020
Complete List of Authors:	Wright, Nicola; University of Nottingham, School Health Sciences<br>Hadziosmanovic, Emina; University of Nottingham, School of Health Sciences<br>Dang, Minh; University of Nottingham, School of Politics and International Relations; Survivor Alliance<br>Bales , Kevin; University of Nottingham, School of Politics and International Relations<br>Brookes, Caroline; Nottinghamshire Healthcare NHS Foundation Trust, Emergency Preparedness Resilience & Response (EPRR)<br>Jordan, Melanie; University of Nottingham, School of Sociology and Social Policy<br>Slade, Mike; University of Nottingham School of Health Sciences, Research Advisory Board, Lived Experience ; Survivor Alliance
**Primary Subject Heading**:	Qualitative research
Secondary Subject Heading:	Qualitative research, Research methods
Keywords:	Adult psychiatry < PSYCHIATRY, QUALITATIVE RESEARCH, Anxiety disorders < PSYCHIATRY

SCHOLARONE™
Manuscripts

I, the Submitting Author has the right to grant and does grant on behalf of all authors of the Work (as defined in the below author licence), an exclusive licence and/or a non-exclusive licence for contributions from authors who are: i) UK Crown employees; ii) where BMJ has agreed a CC-BY licence shall apply, and/or iii) in accordance with the terms applicable for US Federal Government officers or employees acting as part of their official duties; on a worldwide, perpetual, irrevocable, royalty-free basis to BMJ Publishing Group Ltd ("BMJ") its licensees and where the relevant Journal is co-owned by BMJ to the co-owners of the Journal, to publish the Work in this journal and any other BMJ products and to exploit all rights, as set out in our licence.

The Submitting Author accepts and understands that any supply made under these terms is made by BMJ to the Submitting Author unless you are acting as an employee on behalf of your employer or a postgraduate student of an affiliated institution which is paying any applicable article publishing charge ("APC") for Open Access articles. Where the Submitting Author wishes to make the Work available on an Open Access basis (and intends to pay the relevant APC), the terms of reuse of such Open Access shall be governed by a Creative Commons licence – details of these licences and which Creative Commons licence will apply to this Work are set out in our licence referred to above.

Other than as permitted in any relevant BMJ Author's Self Archiving Policies, I confirm this Work has not been accepted for publication elsewhere, is not being considered for publication elsewhere and does not duplicate material already published. I confirm all authors consent to publication of this Work and authorise the granting of this licence.

For peer review only

Title: Mental Health Recovery for Survivors of Modern Slavery: Grounded Theory Study Protocol

Authors: Wright N, Hadziosmanovic E, Dang M, Bales K, Brookes C, Jordan M, Slade M and the Lived Experience Research Advisory Board.

Affiliations:

Dr Nicola Wright, University of Nottingham

Dr Emina Hadziosmanovic, University of Nottingham

Miss Minh Dang, University of Nottingham and Survivor Alliance

Professor Kevin Bales, University of Nottingham

Mrs Caroline Brookes, Nottinghamshire Healthcare NHS Foundation Trust

Dr Melanie Jordan, University of Nottingham

Professor Mike Slade, University of Nottingham

Lived Experience Research Advisory Board, Survivor Alliance

Corresponding Author:

Dr Nicola Wright, School of Health Sciences, University of Nottingham

Address: Queen's Medical Centre, Nottingham, NG7 2UH

Email: Nicola.wright@nottingham.ac.uk

For peer review only

For peer review only - http://bmjopen.bmj.com/site/about/guidelines.xhtml

Abstract

Introduction

Slavery and human trafficking are crimes involving the violation of human rights and refer to exploitative situations where an individual cannot refuse or leave due to threats, coercion or abuse of power. Activities involving slavery include forced labour exploitation, forced sexual exploitation, forced marriage and servitude. Epidemiological studies show high levels of mental health need and poor provision of appropriate support for survivors. What mental health recovery means to victims/survivors and how it could be promoted is under-researched.

Methods and Analysis

A grounded theory study based on individual interviews will be undertaken. Survivors from across the UK will be identified and recruited from Non-Governmental Organisations (NGOs) and via social media. As per grounded theory methodology, data collection and analysis will be undertaken concurrently and recruitment will continue until theoretical saturation is reached. It is anticipated that approximately 30 participants will be recruited. Interviews will be audio recorded, transcribed verbatim and uploaded to NVivo 11. The constant comparative method will be used to analyse the data, in order to produce a theoretical framework for mental health recovery which is grounded in the experiences of survivors.

Ethics and Dissemination

Ethical approval has been obtained from the Faculty of Medicine and Health Sciences Ethics Committee at the University of Nottingham. The findings of the study will be disseminated to academic, professional, and survivor-based audiences to inform future policy developments and the provision of mental health recovery support to this population.

Article Summary

Strengths and limitations of this study

- The study has been co-developed with survivors of modern slavery.
- A lived experience advisory board will facilitate the involvement of survivor voices throughout the research process.
- Survivors who do not speak English will still have the opportunity to take part in the study, through the use of interpreters.
- Modern slavery survivors are not a homogenous population and the recruitment strategy may limit the relevance of the findings to particular sub-groups.
- The theoretical framework will be developed based on a UK sample. This may limit applicability to other contexts.

For peer review only - http://bmjopen.bmj.com/site/about/guidelines.xhtml

Introduction

Slavery and human trafficking are crimes involving the violation of human rights and refer to exploitative situations where an individual cannot refuse or leave due to threats, coercion or abuse of power[1,2]. Activities involving slavery include forced labour exploitation, forced sexual exploitation, forced marriage and servitude. In 2016, it was estimated that approximately 40.3 million people are currently in slavery worldwide[3]. Predicting the future prevalence of slavery is problematic. Globally, the number of vulnerable migrants at risk of trafficking is increasing, with an estimated 65 million people forcibly displaced globally in 2015[4].

In the United Kingdom (UK), research conducted by the Home Office in 2014[5] estimated that there were between 10,000 and 13,000 victims of modern slavery in the UK. However, the Home Office in 2017 has argued that this is the "*tip of the iceberg*" [6]. Due to fear of arrest, deportation or ill treatment by the police, many survivors choose not to access the support provided through official channels, e.g. the National Referral Mechanism (NRM) in the UK[7]. Therefore, official figures do not take into account the unknown numbers of people who are not reported or who continue to live in slavery. Despite these limitations, official statistics do highlight that more people are being freed and are therefore in need of support. The NRM 2019 End of Year Summary reports a 52% increase in referrals compared to 2018[8]. Official statistics also demonstrate that the survivor population within UK is diverse. Survivors originate from 130 different countries and the three most common nationalities are British, Albanian and Vietnamese[8]. In 2019, just over half of NRM referrals were exploited as adults whereas 43% were minors.

Modern slavery (in the UK context this term encompasses both slavery and human trafficking) presents a significant public health concern, and disproportionately affects vulnerable individuals, such as young people, migrants and those living in poverty[9, 10]. Survivors have often experienced extreme violence and psychological abuse [11]. Addressing the mental health needs of this population is therefore now part of anti-trafficking policies in the UK such as the Modern Slavery Act [12], as well as internationally, for example in the Palmero Protocols [13]. Yet, the provision of evidence-based mental health support for this population is one of the largest gaps in both the national and global anti-slavery response [14].

The literature that is available, has predominantly focussed on the mental health needs of women, including those trafficked for sexual exploitation in South or South East Asia and to a lesser extent Europe [15]. Abas and colleagues [16] identified that for women who returned home from the UK to Moldova after being sex trafficked, 55% continued to meet the diagnostic criteria for depression and PTSD six months after their return. Data from high income countries (including the UK), for men and those who have experienced forced labour, is sparse [15].

Despite the emerging evidence base demonstrating high levels of mental distress, tailored support is often found to be insufficient and inadequate leading to high levels of unmet need [17]. When survivors do make contact with mental health services this is frequently via adverse routes, for example detention under the Mental Health Act 1983/2007. Within health care more generally, it is estimated that one in eight members of NHS staff have had contact with survivors [18]. However, health care

For peer review only - http://bmjopen.bmj.com/site/about/guidelines.xhtml

providers report that they feel unprepared to manage and support survivors. For example in the PROTECT studies[18] NHS staff reported not knowing what questions to ask people who may have experience modern slavery and 78% believed they had insufficient training to assist trafficked people.

The limited evidence available from the UK highlights a complex picture in relation to the mental health needs of survivors. High prevalence rates of depression, anxiety, PTSD and suicidal ideation have been identified [7], as well as unmet needs in relation to hearing voices. [7]. For young people who have been trafficked, ADHD and adjustment disorders are common [19]. Whilst these studies have highlighted the level of mental health need within the population, what is not clear is how individuals perceive/experience mental health; what resources they have to overcome their adverse experiences; and what support or interventions would be helpful to facilitate post-traumatic growth and recovery[14].

Post Traumatic Growth (PTG) is defined as the individuals' psychological development after an adverse incident [20]. Whilst traditional theories of resilience emphasise the importance of a return to normal after a traumatic event [21, 22], PTG recognises that adversity can lead to significant changes, some of which may be experienced as positive changes [20]. Rather than a problem or illness orientated position, PTG emphasises the active role of the individual in processing the event and constructing a new identity in the aftermath of trauma [23]. More recent work has started to apply the theory of PTG to clinical populations, for example those with psychosis [24]. Through this personal growth process, the strengths and resources of the individual are emphasised, and positive adaptions learnt.

Within this context, modern slavery survivors are seen as active participants in their care, with resources and strengths which can be utilised for mental health recovery [25]. This can include factors within the individual, such as coping strategies, and also those external to them, for example social networks. Recovery in this context is therefore a subjective experience and has been defined as “a deeply personal, unique process of changing one’s attitudes, values, feelings, goals, skills and/or roles” and as “a way of living a satisfying, hopeful and contributing life even within the limitations caused by illness” [26].

Recovery has become the underpinning discourse for mental health service provision globally [26] and in England [27]. A key insight from this perspective is that living well involves less emphasis on symptom amelioration, and a stronger focus on addressing psychological and social needs, supporting self-management and building individual and community resilience [28]. This understanding is relevant to work with survivors of modern slavery, who may have mental health symptoms (e.g. those related to trauma) and also a range of other psychological (e.g. self-identity), social (e.g. anticipated and experienced discrimination) and cultural challenges (e.g. dislocation).

The aim of this study is to develop a theoretically informed understanding of mental health recovery for survivors of modern slavery within the UK. The objectives are: (1) To understand the mental health needs and strengths of modern slavery survivors; (2) To explore with modern slavery survivors the concept of mental health recovery; what it means to them; and how it could be promoted; and (3) To construct a theoretically

For peer review only - http://bmjopen.bmj.com/site/about/guidelines.xhtml

informed framework for mental health recovery, based on the experiences of modern slavery survivors, which can inform a future intervention.

Methods and Analysis

Design

A qualitative study informed by grounded theory methodology based on individual, semi-structured interviews with modern slavery survivors is proposed. Widely used in health and social sciences, grounded theory is an inductive methodology which facilitates the development of a theoretical framework grounded in the experiences of participants[27].

Setting

Participants will be recruited from relevant community based Non-Governmental Organisations (NGOs). This will include both NRM and non-NRM provider organisations.

Sample

Eligibility criteria:

(1) Self-identify or be identified by an NGO as a survivor of modern slavery. Self-identifying involves describing being in situations or having experiences that align with modern slavery, even if the term 'modern slavery' is not used by the participant.
(2) Be currently in the UK
(3) Be aged over 18 years.
(4) Have exited slavery for more than 12 months.
(5) Be able to participate in an interview process conducted in English, either with or without the assistance of an interpreter.
(6) Be able and willing to discuss issues related to mental health/distress. A formal diagnosis of a mental health problem is not required, although potential participants should have one of: (a) self-reported clinical diagnosis; (b) recent (in the last two years) primary or secondary mental health service use; OR (c) self-reported recent (in the last two years) experience of mental distress

Procedures

Aligned with the study design, theoretical sampling will be used. This is an iterative process whereby data collection and analysis occur simultaneously, allowing insights from preliminary analysis to inform the recruitment of future participants. This will occur until data saturation is reached, i.e. no new insights or themes are identified [29]. After an initial sample of participants have been recruited and their data analysed, future sampling will address potentially any of the following:

(1) Gaps in relation to participant demographics (for example gender, age, ethnicity, experience of a particular form of slavery).
(2) Emergent theoretical concepts, for example to understand or elucidate a particular insight in more detail

For peer review only - http://bmjopen.bmj.com/site/about/guidelines.xhtml

(3) Any explanatory gaps between the concepts identified, for example to demonstrate the relationship between two different concepts

If necessary, theoretical sampling will be supplemented with snowball sampling techniques, whereby participants are asked to identify from within their networks other individuals to approach for interview. It is acknowledged that snowball sampling could potentially narrow the demographic focus of participants and so will only be used once other recruitment methods have been exhausted. The nature of theoretical sampling and achieving data saturation means that determining in advance the precise sample size is problematic. Previous grounded theory studies suggest that 30 interviews is likely to be sufficient to achieve saturation [30, 31]. Therefore, this study will recruit, at least, 30 modern slavery survivors or until data saturation is achieved.

In-depth, semi-structured interviews will be conducted with participants. Distress protocols have been put in place in order to protect the research participants, the researcher undertaking the interviews, interpreters and transcribers. The researcher will receive relevant training to ensure they have the skills required. They will also receive monthly clinical supervision to assist with the processing of any difficult material or interviews. Participants will be made aware that if they make disclosures which indicate they are at risk this may need to be escalated to the appropriate authorities. Signposting materials to support organisations will also be available to participants should these be required.

Where necessary, independent NHS-approved interpreters will be used during the interviews and written materials translated. The interview schedules were developed in collaboration with the Lived Experience Advisory Board and informed by the World Health Organisation recommendations for interviewing trafficked women [32]. As per the iterative nature of grounded theory, the interview schedules will continue to evolve during the course of the project to address emergent concepts and ideas with participants.

Recruitment of participants will take place via relevant NGOs who provide support to survivors of modern slavery, and by advertising the study widely through social media. Twitter will be the main platform used for recruitment. The recruitment poster will be shared and Direct Messaging to the researcher and principal investigator made possible in the event of any questions. The research team will work with the NGOs involved to distribute study information and identify people who meet the eligibility criteria. Individuals who are interested in participating in the study will be asked to contact the research team by either phone or email. After explaining the study purpose (face to face, by phone or online depending on the participant's preference), including the voluntary and anonymous nature of participation, and distributing the information sheet, informed written consent will be obtained. All written documentation will be provided to participants in the language of their choice. The interviews (approximate duration 60 minutes) will be audio-recorded and transcribed verbatim. They will be conducted at a time of the participants choosing, whenever possible as a face-to-face interview in a place which assures the participant of safety, privacy and comfort. Where this is not possible, interviews will be offered via telephone or video call and the participant will be able to choose based on personal preference which they would like. Audio recordings using an encrypted device will be made for these interviews too.

For peer review only - http://bmjopen.bmj.com/site/about/guidelines.xhtml

Data Analysis

Interview transcripts will be uploaded to NVivo 11 software for analysis. The constant comparative method will be used to generate successively more abstract concepts and theories through the inductive coding of individual transcripts [33]. The codes identified will then be compared against those generated in other transcripts. This will identify both consistency and any divergence in the theoretical framework being constructed. Finally, the emergent theoretical framework will be compared against existing models and theories of recovery in mental health, such as CHIME (connectedness, hope, identity, meaning and empowerment) [34]. They will assist with the identification of both what is novel for the survivor population and also what is similar and adapted from other populations.

Patient and Public Involvement

Patient and public involvement is a core component of the project. As per guidelines [35] survivors are engaged in the five key stages of the research process:

(1) Design of the research.
(2) Development of the grant application/pre-protocol work.
(3) Undertaking or managing the research.
(4) Analysis of data.
(5) Dissemination of findings.

A survivor led, lived experience research advisory board has been developed in collaboration with the Survivor Alliance (www.survivoralliance.org), a non-profit organisation that unites and empowers survivors of slavery and human trafficking worldwide. The role of the advisory board will be to provide a survivor perspective on all aspects of the study, including language and format of consent forms and information sheets, recruitment strategy, data analysis and dissemination. The group will meet six times during the course of the project.

Ethics and Dissemination

Ethical approval for the study was obtained from the Faculty of Medicine and Health Sciences, University of Nottingham in December 2019 (reference number 436-1912). The main ethical concern of the project is the potential to cause emotional or psychological distress to participants, if they recount their traumatic experiences. The project aims to minimise this by not specifically asking questions relating to the original trauma or trafficking experience. Instead, the interview will focus on mental health recovery and what this means to individuals. It is acknowledged that individuals may choose to recount their difficult experiences and if they do so, the distress protocol (Supplementary Information 1) will be adhered to. All the questions in the interview schedule have been reviewed and approved by the Lived Experience Research Advisory Board (RAB) who felt there was an important distinction to be made between survivors choosing to bring up topics themselves as opposed to being asked to talk about their trafficking experiences. Initial drafts of questions were amended according to the advice of RAB members. They did not feel that the final questions included in the interview schedule would be triggering for other survivors.

For peer review only - http://bmjopen.bmj.com/site/about/guidelines.xhtml

Members of RAB also felt it important to explicitly note in the interview schedule that mental health support is not available as part of survivor's participation in the study; the researcher is not able to provide legal or mental health support. However, individuals will be made aware that they can contact the researcher to find out about mental health services in their local area. Due to the study being nation-wide, this information cannot be included on the debriefing information sheet. All participants will receive details of national support available (e.g., Samaritans helpline).

Participants will remain anonymous, their names (and any other identifiable details) will be replaced by a pseudonym at the point of transcription. Individuals' identities will not be disclosed in the final report or any subsequent publications. Secure electronic and paper based filing systems for both the recordings and transcriptions will be established. All study related data will be kept for a minimum of 7 years after the study end date.

The findings of the research will be disseminated to academic, professional, survivor, and policy audiences via written publication, verbal presentations and knowledge sharing events.

References

1) Williamson, V, et al. Responding to the needs of trafficked people: A qualitative study of professionals in England and Scotland. **Health and Social Care in the Community** 2020; 28: 173 – 181.

2) International Labour Organisation, **Methodology of the global estimates of modern slavery: Forced labour and forced marriage.** The International Labour Office: Geneva. 2017.

3) Global Slavery Index, **The Global Slavery Index 2018.** [online] Accessed on: 3rd March 2020 at www.globalslaveryindex.org.

4) United Nations Office on Drugs and Crime, **Global Report on Trafficking in Persons.** United Nations: New York. 2016.

5) Silverman, B, **Modern Slavery: An Application of Multiple Systems Estimation.** Home Office: London. 2014.

6) Home Office, **2017 UK Annual Report on Modern Slavery.** [online] Accessed on: 3rd March 2020 at https://www.gov.uk/government/publications/2017-uk-annual-report-on-modern-slavery.

7) Oram, S, *et al.* Human trafficking and health: A survey of male and female survivors in England. **American Journal of Public Health** 2016; 106: 6 1073-1078.

8) Home Office, **2019 UK Annual Report on Modern Slavery.** HMSO: London. 2019.

For peer review only - http://bmjopen.bmj.com/site/about/guidelines.xhtml

https://assets.publishing.service.gov.uk/government/uploads/system/uploads/attachment_data/file/876646/national-referral-mechanism-statistics-uk-end-of-year-summary-2019.pdf

9) Zimmerman, C. and Kiss, L. Human trafficking and exploitation: A global health concern. **PLOS Medicine** 2017; 14: 11 e1002437.

10) Such, E, *et al*. Modern slavery and public health: a rapid evidence assessment and an emergent public health approach. **Public Health.** 2020; 180: 168-179.

11) International Organisation of Migration, **Report of the Counter-Trafficking Unit: Return and Reintegration Project.** International Organisation for Migration: Brussels. 2002.

12) The UK Government, **Modern Slavery Act.** HMSO: London. 2015.

13) United Nations, **Protocol to Prevent, Suppress and Punish Trafficking in Persons, Especially Women and Children, Supplementing the United Nations Convention Against Transnational Organised Crime**. New York: UNODC. 2000.

14) Katona, C, *et al.* **Addressing Mental Health Needs in Survivors of Modern Slavery: A Critical Review and Research Agenda.** Helen Bamber Foundation: London. 2015.

15) Oram, S, *et al.* International law, national policymaking and the health of trafficked people in the UK. **Health and Human Rights** 2011; 13 (2): E3-16.

16) Abas, M, *et al.* Risk factors for mental disorders in women survivors of human trafficking: A historical cohort study. **BMC Psychiatry** 2013; 13: 204.

17) Such, E, and Salway, S. **Research Analysis: Modern Slavery and Public Health.** Public Health England: London. 2017.

18) Oram, S, *et al.* **PROTECT: Provider Responses Treatment and Care for Trafficked People. Final Report for the Department of Health Policy Research Programme.** Department of Health: London. 2015.

19) Stanley, N, *et al*. The health needs and healthcare experiences of young people trafficked into the UK. **Child Abuse and Neglect** 2016; 59: 100-110.

20) Tedeschi, RG. and Calhoun, LG. **Posttraumatic Growth: Conceptual Foundation and Empirical Evidence.** Lawrence Erlbaum Associates: Philadelphia. 2004.

21) Higgins, G.O. **Resilient Adults: Overcoming a Cruel Past.** Jossey-Boss: San Francisco. 1994.

22) Wollin, S.J. and Wollin, S. **The Resilient Self: How Survivors of Troubled Families Rise Above Adversity.** Villard Books: New York. 1993.

For peer review only - http://bmjopen.bmj.com/site/about/guidelines.xhtml

23)Carver, C. Resilience and thriving: Issue, models and linkages. **Journal of Social Issues.** 2010; 54: 245-266.

24)Slade, M, *et al.* Post-traumatic growth in mental health recovery: qualitative study of narratives. **British Medical Journal.** 2019; Doi: 10.1136/bmjopen-2019-029342.

25)Repper, J. and Perkins, R. **Social Inclusion and Recovery: A Guide for Mental Health Practice.** Bailiere Tindall: London. 2003.

26)World Health Organisation **Mental Health Action Plan 2013-2020.** WHO Press: Geneva. 2013.

27)Department of Health **No Health without Mental Health: A Cross Government Mental Health Outcomes Strategy for All Ages.** Department of Health: London. 2011.

28)Slade, M, *et al.* Uses and abuses of recovery: Implementing recovery-orientated practices in mental health systems. **World Psychiatry** 2014; 13: 12-20.

29)Charmaz, K. **Constructing Grounded Theory.** 2nd Edition. SAGE: New York. 2014.

30)Dworkin, S.L. Sample size policy for qualitative studies using in-depth interviews. **Archives of Sexual Behaviour** 2012; 41: 6 1319-1320

31)Palmquist, L, *et al.* Protocol: A grounded theory study of 'recovery': Perspectives of adolescent users of mental health services. **BMJOpen** 2017; 7:e015161. doi: 10.1136/bmjopen-2016-01516

32)Zimmerman, C. **WHO Ethical and Safety Recommendations for Interviewing Trafficked Women.** WHO Press: Geneva. 2003.

33)Glaser, B.G. and Strauss, A. L. **The Discovery of Grounded Theory**. Aldine: Chicago. 1967.

34)Leamy, M.E, *et al.* A conceptual framework for personal recovery in mental health: Systematic review and narrative synthesis. **British Journal of Psychiatry** 2014; 199: 445-452.

35)NIHR **Patient and Public Involvement in Health and Social Care Research: A Handbook for Researchers.** NIHR: London. 2014.

For peer review only

For peer review only - http://bmjopen.bmj.com/site/about/guidelines.xhtml

Contribution

NW and MD conceived of the research idea and with KB, CB, MJ and MS designed the study and secured funding. EH wrote the initial draft of this paper. LERAB contributed to the development of the research materials. All authors have read, edited and revised the paper.

Funding Statement

This study is funded by the National Institute for Health Research (NIHR) [Research for Patient Benefit, project reference PB-PG-1217-20036]. Mike Slade acknowledges the support of Centre for Mental Health and Substance Abuse, University of South-Eastern Norway and the NIHR Nottingham Biomedical Research Centre. The views expressed are those of the authors and not necessarily those of the NIHR or the Department of Health and Social Care.

Competing Interests Statement

There are no declarations of competing interests

Patient Consent for Publication

Not required.

Ethics Approval

The study has been approved by the Faculty of Medicine and Health Sciences Ethics Committee at the University of Nottingham.

For peer review only

•Distress

- A participant indicates that they are experiencing distress. **OR**
- A participant exhibits behaviours suggestive that the interview is causing distress. For example crying, shaking, agitation.

•Stage 1 response

- The researcher stops the interview.
- The participant is offered immediate support, time out etc..
- The researcher asks the participant how they are feeling, what their thoughts are and whether they feel safe.

•Review

- If the participant feels able to carry on resume the interview.
- If the participant is unable to carry on go to stage 2 response.

•Stage 2 response

- Discontinue interview.
- The participant is encouraged to seek support from their usual contact points. This could be a caseworker/therapist at an NGO, their GP, a local authority worker or mental health team. **OR**
- With consent offer for a member of the research team to make contact with the participant usual support system. **OR**
- With consent seek advice or further support from the participants main contact point.

•Follow up

- If participant consents follow up with a courtesy phone call. **OR**
- Encourage the participant to call if s/he experiences increasing levels of distress in the hours/days post interview.
- All participants to be given an information sheet with relevant organisations contact details and places for support (translated if necessary).

•Distress Protocol 1: The protocol for managing participant distress in the context of a research interview

•Pre-data collection

- The potential physical and psychological impact on the researcher of the participants description of their life events should be considered.
- In collaboration with the PI and wider research team a decision should be made as to how many interviews could be undertaken in a week.
- The potential for emotional exhaustion should be acknowledged.
- A mechanism for debriefing and clinical supervision should be established to provide support during data collection and analysis.

• Data collection stage

- Consider where interviews should take place to ensure support is available . Also take into account the University of Nottingham Lone Worker Policy to ensure safety.
- Debriefing to be available with the PI or another member of the research team immediately after an interview has been completed if required.
- Monthly clinical supervision to be arranged with a member of staff external to the research team to facilitate reflection and processing of events.
- Encourage the researcher to keep a reflective diary which records thoughts and feeling which could become part of research fieldnotes if appropriate.

•Analysis

- For the researcher to be aware that engaging in data analysis of potentially distressing material could be emotionally triggering.
- Schedule monthly clinical supervision during this period of time to discuss emerging situations, encourage reflection and processing of events.
- Access debriefing as required with a member of the research team.
- Encourage the researcher to keep a reflective diary which records thoughts and feeling which could become part of research fieldnotes if appropriate.

•Follow up

- Encourage the researcher to seek counselling and other support in addition to debriefing and clinical supervision should s/he experience increased distress as a result of engagement in research activities.

•Distress Protocol 2: The protocol for managing distress in the context of research interview management.

•Pre-data collection

- The transcriber should be considered in any research proposal, with a clear indication of how this person will be provided with an emotionally safe working environment which also maintains the quality of the research product.

•Ethical •considerations

- Consideration is given to the support needs of the transcriber as part of the ethical review process.
- The transcriber should be informed of the nature of the research and the type of data to be collected.

•Pre- •transcription

- The transcriber is alerted prior to commencing transcription of any potentially challenging or difficult interviews.
- The transcriber has access to regular debriefing with the PI or another member of the research team should this be required.

•During transcription

- Prompt access to an appropriate person for crisis debriefing to be facilitated should this be required.
- The transcriber is able to terminate their involvement in the transcription process (either of an individual interview or from the whole project) if deemed necessary with appropriate support given to resolve any personal issues that arise as consequence of this work.
- Encourage the transcriber to keep a reflective diary to record their thoughts and feelings which may become part of fieldnotes if appropriate.

•Follow up

- If the transcriber consents, follow up with a courtesy call. **OR**
- Encourage the transcriber to call if s/he experiences increased distress in the hours/days following transcription.

•Distress Protocol 3: The protocol for managing distress in the context of research interview transcription.

For peer review only - http://bmjopen.bmj.com/site/about/guidelines.xhtml

•Pre-data collection

•The interpreter should be considered in any research proposal, with a clear indication of how this person will be provided with an emotionally safe working environment which also maintains the quality of the research product.

•Ethical •consideration s

•Consideration is given to the support needs of the interpreter as part of the ethical review process.

•The interpreter should be informed of the nature of the research and the type of data to be collected.

•Data collection

•The interpreter is alerted to any potentially challenging interviews which have been scheduled in advance.

•Debriefing to be available with the PI or another member of the research team immediately after an interview has been completed if required.

•Encourage the interpreter to keep a reflective diary which records thoughts and feeling which could become part of research fieldnotes if appropriate.

•Follow up

•If the interpreter consents, follow up with a courtesy call. **OR**

•Encourage the interpreter to call if s/he experiences increased distress in the hours/days following transcription.

•Distress Protocol 4: The protocol for managing distress in the context of research interview interpretation.

•Supporting References

- Draucker CB, Martsolf DS & Poole C
  - (2009) Developing distress protocols for
  - research on sensitive topies. **Archives**
  - **of Psychiatric Nursing** 23 (5) 343-350.
- Gregory D, Russell C & Phillips L (1997)
  - Beyond textual perfection: transcribers
  - as vulnerable persons. **Qualitative**
  - **Health Research** 7 (2) 294-300.
- Haigh C & Witham G (2015) **Distress**
  - **Protocol for Qualitative Data**
  - **Collection.** Manchester Metropolitan
  - University.
- McCosker H, Barnard A & Gerber R
  - (2001) Undertaking sensitive research:
  - Issues and strategies for meeting the
  - safety needs of all. **Forum: Qualitative**
  - **Social Research** 2 (1).
